# 1-(2,4-Dinitro­phenyl)-3-(4-methyl­phenyl)-4-phenyl­sulfanyl-1*H*-pyrazole

**DOI:** 10.1107/S160053680803122X

**Published:** 2008-10-11

**Authors:** P. Ramesh, Ramaiyan Venkatesan, Ramaiyan Manikannan, S. Muthusubramanian, M. N. Ponnuswamy

**Affiliations:** a Department of Physics, Presidency College (Autonomous), Chennai 600 005, India; b Department of Organic Chemistry, School of Chemistry, Madurai Kamaraj University, Madurai 625 021, India; c Centre of Advanced Study in Crystallography and Biophysics, University of Madras, Guindy Campus, Chennai 600 025, India

## Abstract

In the title compound, C_22_H_16_N_4_O_4_S, the dihedral angles between the pyrazole ring and the pendant aromatic rings are 26.2 (1), 41.1 (1) and 89.5 (1)°. In the crystal structure, an intermolecular C—H⋯N bond helps to establish the packing. A short C⋯C contact of 3.110 (12) Å is observed between the C atom of the pyrazole CH group and one of the α-C atoms of the 4-methyl­phenyl ring.

## Related literature

For related literature, see: Baraldi *et al.* (1998 ▶); Beddoes *et al.* (1986 ▶); Bruno *et al.* (1990 ▶); Cottineau *et al.* (2002 ▶); Londershausen (1996 ▶); Bernstein *et al.* (1995 ▶); Chen & Li (1998 ▶); Cordell (1981 ▶); Jin *et al.* (2004 ▶); Smith *et al.* (2001 ▶).
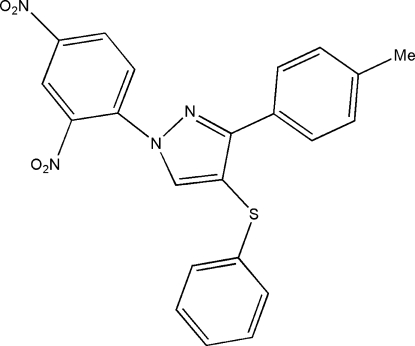


## Experimental

### 

#### Crystal data

C_22_H_16_N_4_O_4_S
*M* *_r_* = 432.45
Monoclinic, 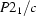

*a* = 7.3802 (3) Å
*b* = 26.6996 (11) Å
*c* = 10.6691 (4) Å
β = 106.733 (2)°
*V* = 2013.31 (14) Å^3^
*Z* = 4
Mo *K*α radiation
μ = 0.20 mm^−1^
*T* = 293 (2) K
0.25 × 0.21 × 0.19 mm

#### Data collection

Bruker APEXII CCD area-detector diffractometer
Absorption correction: multi-scan (*SADABS*; Sheldrick, 2001 ▶) *T*_min_ = 0.951, *T*_max_ = 0.963
26409 measured reflections
5987 independent reflections
4104 reflections with *I* > 2σ(*I*)
*R*_int_ = 0.030

#### Refinement

*R*[*F* ^2^ > 2σ(*F* ^2^)] = 0.050
*wR*(*F* ^2^) = 0.154
*S* = 1.05
5987 reflections
282 parameters
1 restraint
H-atom parameters constrained
Δρ_max_ = 0.38 e Å^−3^
Δρ_min_ = −0.27 e Å^−3^

### 

Data collection: *APEX2* (Bruker, 2004 ▶); cell refinement: *SAINT* (Bruker, 2004 ▶); data reduction: *SAINT*; program(s) used to solve structure: *SHELXS97* (Sheldrick, 2008 ▶); program(s) used to refine structure: *SHELXL97* (Sheldrick, 2008 ▶); molecular graphics: *ORTEP* (Farrugia, 1997 ▶); software used to prepare material for publication: *SHELXL97* and *PLATON* (Spek, 2003 ▶).

## Supplementary Material

Crystal structure: contains datablocks global, I. DOI: 10.1107/S160053680803122X/bt2786sup1.cif

Structure factors: contains datablocks I. DOI: 10.1107/S160053680803122X/bt2786Isup2.hkl

Additional supplementary materials:  crystallographic information; 3D view; checkCIF report

## Figures and Tables

**Table 1:** Hydrogen-bond geometry (Å, °)

*D*—H⋯*A*	*D*—H	H⋯*A*	*D*⋯*A*	*D*—H⋯*A*
C5—H5⋯N2^i^	0.93	2.44	3.2847 (18)	152

Symmetry code: (i) 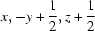.

**Fig. 1.**
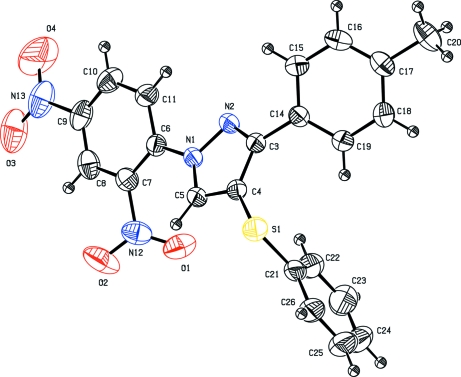
Perspective view of the molecule showing the thermal ellipsoids are drawn at 50% probability level. The H atoms are shown as small spheres of arbitrary radii.

**Fig. 2.**
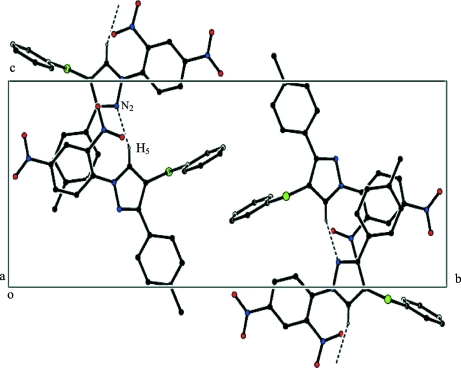
The packing diagram viewed down c axis.

## supplementary crystallographic information

### Comment

Pyrazole derivatives have been reported to possess significant antiarrhythmic and sedative (Bruno *et al.*, 1990), hypoglycemic (Cottineau *et al.*, 2002), antiviral (Baraldi *et al.*, 1998), and pesticidal (Londershausen,1996) activities.

The *ORTEP* plot of the structure is shown in Fig. 1. The pyrazole ring adopts planar conformation. The sum of the angles at N1 of the pyrazole ring (359.55°) is in accordance with *sp*^2^ hybridization (Beddoes *et al.*, 1986). The C—N bond lengths in the pyrazole ring are 1.319 (2) and 1.352 (2) Å, which are shorter than a C—N single bond length of 1.443 Å, but longer than a double bond length of 1.269 Å (Jin *et al.*, 2004), indicating electron delocalization. The pyrazole ring A and phenyl sulfide ring D are orthogonal with the inter-ring dihedral angle of 89.54 (10)°, whereas the dinitrophenyl and methylphenyl rings are twisted from the pyrazole ring as can be seen from the dihedral angles of 26.18 (10)° and 41.12 (10)°, respectively.

The crystal structure is stabilized by C—H···N types of intra and intermolecular interactions in addition to van der Waals forces. Atom C5 in the molecule at (*x*, *y*, *z*) donates a proton to atom N2 at (*x*, -1/2 - *y*, 1/2 + *z*) to form a one dimensional C4 chain running along *c* axis (Fig. 2.). Short intermolecular contacts are observed between the atoms C5 and C15 (3.11 Å).

### Experimental

A mixture of 1-(4-Methylphenyl)-2-(phenylsulfanyl)-1-ethanone 1-(2,4-dinitrophenyl)hydrazone (0.001 mole) dissolved in dimethylforamide (5 ml) in a 30 ml conical flask was allowed to cool in ice with stirring. To this stirred solution, phosphorus oxychloride (0.008 mole) was added dropwise and the mixture was subjected to microwave irritation for 60 sec. under 40% power with pulse rate 15 sec. The reaction was monitored by TLC and after completion of the reaction, the reaction mixture was poured onto crushed ice. The solid was suction filtered and washed with plenty of water. The final product 1-(2,4-Dinitrophenyl)-3-(4-methylphenyl)-1*H*-4- pyrazolyl phenyl sulfide was purified by column chromatography silica gel (60–120 mesh) using petroleum ether- ethyl acetate as eluent.

### Refinement

H atoms were positioned geometrically (C—H=0.93–0.96 Å) and allowed to ride on their parent atoms, with *U*_iso_(H) = 1.5 *U*_eq_(C) for methyl H and *U*_iso_(H) = 1.2 *U*_eq_(C) other H atoms. The components of the anisotropic displacement parameters  of C24 and C25 in the direction of the bond between them were restrained to be equal within an effective standard deviation of 0.01.

### Crystal data

**Table:**

C_22_H_16_N_4_O_4_S	*F*(000) = 896
*M**_r_* = 432.45	*D*_x_ = 1.427 Mg m^−^^3^
Monoclinic, *P*2_1_/*c*	Mo *K*α radiation, λ = 0.71073 Å
Hall symbol: -P 2ybc	Cell parameters from 5342 reflections
*a* = 7.3802 (3) Å	θ = 1.5–30.3°
*b* = 26.6996 (11) Å	µ = 0.20 mm^−^^1^
*c* = 10.6691 (4) Å	*T* = 293 K
β = 106.733 (2)°	Block, colorless
*V* = 2013.31 (14) Å^3^	0.25 × 0.21 × 0.19 mm
*Z* = 4	

### Data collection

**Table:**

Bruker APEXII CCD area-detector diffractometer	5987 independent reflections
Radiation source: fine-focus sealed tube	4104 reflections with *I* > 2σ(*I*)
graphite	*R*_int_ = 0.031
ω and φ scans	θ_max_ = 30.3°, θ_min_ = 1.5°
Absorption correction: multi-scan (SADABS; Sheldrick, 2001)	*h* = −10→10
*T*_min_ = 0.951, *T*_max_ = 0.963	*k* = −36→37
26409 measured reflections	*l* = −14→15

### Refinement

**Table:**

Refinement on *F*^2^	Secondary atom site location: difference Fourier map
Least-squares matrix: full	Hydrogen site location: inferred from neighbouring sites
*R*[*F*^2^ > 2σ(*F*^2^)] = 0.050	H-atom parameters constrained
*wR*(*F*^2^) = 0.154	*w* = 1/[σ^2^(*F*_o_^2^) + (0.0774*P*)^2^ + 0.3656*P*] where *P* = (*F*_o_^2^ + 2*F*_c_^2^)/3
*S* = 1.05	(Δ/σ)_max_ = 0.039
5987 reflections	Δρ_max_ = 0.38 e Å^−^^3^
282 parameters	Δρ_min_ = −0.27 e Å^−^^3^
1 restraint	Extinction correction: SHELXL97 (Sheldrick, 2008), Fc^*^=kFc[1+0.001xFc^2^λ^3^/sin(2θ)]^-1/4^
Primary atom site location: structure-invariant direct methods	Extinction coefficient: 0.0074 (13)

### Special details

**Table:**

Geometry. All e.s.d.'s (except the e.s.d. in the dihedral angle between two l.s. planes) are estimated using the full covariance matrix. The cell e.s.d.'s are taken into account individually in the estimation of e.s.d.'s in distances, angles and torsion angles; correlations between e.s.d.'s in cell parameters are only used when they are defined by crystal symmetry. An approximate (isotropic) treatment of cell e.s.d.'s is used for estimating e.s.d.'s involving l.s. planes.
Refinement. Refinement of *F*^2^ against ALL reflections. The weighted *R*-factor *wR* and goodness of fit *S* are based on *F*^2^, conventional *R*-factors *R* are based on *F*, with *F* set to zero for negative *F*^2^. The threshold expression of *F*^2^ > σ(*F*^2^) is used only for calculating *R*-factors(gt) *etc*. and is not relevant to the choice of reflections for refinement. *R*-factors based on *F*^2^ are statistically about twice as large as those based on *F*, and *R*- factors based on ALL data will be even larger.

### Fractional atomic coordinates and isotropic or equivalent isotropic displacement parameters (Å^2^)

**Table:**

	*x*	*y*	*z*	*U*_iso_*/*U*_eq_	
S1	0.24305 (7)	0.133863 (17)	0.05946 (4)	0.04676 (15)	
O1	0.8025 (2)	0.24070 (6)	0.22748 (16)	0.0669 (4)	
O2	0.8088 (3)	0.29587 (8)	0.37625 (14)	0.0842 (6)	
O3	0.8511 (3)	0.46380 (7)	0.2362 (2)	0.0960 (7)	
O4	0.7256 (4)	0.48434 (8)	0.0364 (3)	0.1256 (9)	
N1	0.4753 (2)	0.25931 (5)	0.00834 (11)	0.0335 (3)	
N2	0.4052 (2)	0.24683 (5)	−0.12163 (11)	0.0347 (3)	
C3	0.3241 (2)	0.20272 (6)	−0.12297 (13)	0.0322 (3)	
C4	0.3385 (2)	0.18664 (6)	0.00663 (14)	0.0347 (3)	
C5	0.4325 (2)	0.22374 (6)	0.08542 (14)	0.0357 (4)	
H5	0.4623	0.2246	0.1762	0.043*	
C6	0.5566 (2)	0.30667 (6)	0.04053 (14)	0.0345 (3)	
C7	0.6824 (2)	0.31920 (7)	0.16180 (15)	0.0391 (4)	
C8	0.7453 (3)	0.36745 (7)	0.19047 (19)	0.0483 (4)	
H8	0.8243	0.3757	0.2728	0.058*	
C9	0.6891 (3)	0.40307 (7)	0.0953 (2)	0.0502 (5)	
C10	0.5710 (3)	0.39244 (7)	−0.0262 (2)	0.0518 (5)	
H10	0.5368	0.4172	−0.0899	0.062*	
C11	0.5037 (3)	0.34446 (6)	−0.05236 (17)	0.0433 (4)	
H11	0.4209	0.3371	−0.1340	0.052*	
N12	0.7691 (2)	0.28207 (7)	0.26313 (15)	0.0524 (4)	
N13	0.7602 (3)	0.45433 (7)	0.1245 (3)	0.0727 (6)	
C14	0.2370 (2)	0.17731 (6)	−0.24803 (14)	0.0339 (3)	
C15	0.1349 (2)	0.20461 (6)	−0.35568 (15)	0.0384 (4)	
H15	0.1154	0.2387	−0.3474	0.046*	
C16	0.0619 (3)	0.18132 (7)	−0.47542 (16)	0.0458 (4)	
H16	−0.0059	0.2001	−0.5470	0.055*	
C17	0.0875 (3)	0.13087 (7)	−0.49080 (16)	0.0461 (4)	
C18	0.1858 (3)	0.10358 (7)	−0.38248 (17)	0.0460 (4)	
H18	0.2027	0.0693	−0.3908	0.055*	
C19	0.2596 (3)	0.12621 (6)	−0.26211 (15)	0.0409 (4)	
H19	0.3245	0.1071	−0.1903	0.049*	
C20	0.0110 (4)	0.10575 (10)	−0.62168 (19)	0.0699 (6)	
H20A	−0.0225	0.0718	−0.6090	0.105*	
H20B	0.1058	0.1060	−0.6672	0.105*	
H20C	−0.0991	0.1234	−0.6723	0.105*	
C21	0.4319 (3)	0.09067 (6)	0.09185 (15)	0.0452 (4)	
C22	0.5983 (3)	0.09830 (8)	0.06220 (19)	0.0548 (5)	
H22	0.6194	0.1282	0.0239	0.066*	
C23	0.7352 (3)	0.06106 (9)	0.0897 (2)	0.0709 (7)	
H23	0.8478	0.0658	0.0686	0.085*	
C24	0.7049 (4)	0.01713 (9)	0.1480 (2)	0.0782 (7)	
H24	0.7973	−0.0077	0.1669	0.094*	
C25	0.5401 (5)	0.01001 (9)	0.1779 (2)	0.0788 (7)	
H25	0.5206	−0.0197	0.2177	0.095*	
C26	0.4027 (4)	0.04593 (7)	0.15001 (19)	0.0621 (6)	
H26	0.2896	0.0405	0.1699	0.075*	

### Atomic displacement parameters (Å^2^)

**Table:**

	*U*^11^	*U*^22^	*U*^33^	*U*^12^	*U*^13^	*U*^23^
S1	0.0607 (3)	0.0369 (2)	0.0481 (2)	−0.0063 (2)	0.0242 (2)	0.00621 (17)
O1	0.0606 (10)	0.0587 (10)	0.0737 (10)	0.0097 (8)	0.0071 (8)	0.0176 (8)
O2	0.0954 (13)	0.1076 (14)	0.0373 (7)	−0.0208 (11)	−0.0005 (8)	0.0040 (8)
O3	0.0905 (14)	0.0649 (11)	0.1282 (17)	−0.0231 (10)	0.0248 (12)	−0.0467 (11)
O4	0.168 (2)	0.0447 (11)	0.149 (2)	−0.0344 (13)	0.0217 (18)	0.0081 (12)
N1	0.0451 (8)	0.0293 (6)	0.0273 (5)	−0.0015 (6)	0.0123 (5)	−0.0012 (5)
N2	0.0440 (8)	0.0342 (7)	0.0267 (6)	−0.0006 (6)	0.0117 (5)	0.0003 (5)
C3	0.0383 (8)	0.0296 (8)	0.0300 (6)	0.0013 (6)	0.0118 (6)	0.0005 (5)
C4	0.0464 (9)	0.0284 (8)	0.0318 (7)	0.0010 (7)	0.0154 (6)	0.0018 (5)
C5	0.0489 (10)	0.0332 (8)	0.0275 (6)	0.0026 (7)	0.0150 (6)	0.0019 (6)
C6	0.0389 (9)	0.0305 (8)	0.0359 (7)	−0.0007 (6)	0.0137 (6)	−0.0020 (6)
C7	0.0380 (9)	0.0421 (9)	0.0380 (8)	−0.0018 (7)	0.0123 (7)	−0.0010 (7)
C8	0.0429 (10)	0.0518 (11)	0.0507 (10)	−0.0090 (8)	0.0142 (8)	−0.0163 (8)
C9	0.0485 (11)	0.0332 (9)	0.0721 (12)	−0.0070 (8)	0.0226 (10)	−0.0095 (8)
C10	0.0588 (12)	0.0331 (9)	0.0629 (11)	−0.0011 (8)	0.0164 (9)	0.0051 (8)
C11	0.0511 (10)	0.0346 (9)	0.0421 (8)	−0.0013 (8)	0.0100 (7)	0.0020 (7)
N12	0.0445 (9)	0.0629 (11)	0.0452 (8)	−0.0081 (8)	0.0055 (7)	0.0086 (7)
N13	0.0709 (13)	0.0410 (10)	0.1077 (17)	−0.0137 (9)	0.0283 (12)	−0.0197 (11)
C14	0.0361 (8)	0.0354 (8)	0.0305 (7)	−0.0019 (7)	0.0101 (6)	−0.0009 (6)
C15	0.0389 (9)	0.0377 (9)	0.0380 (8)	0.0011 (7)	0.0104 (7)	0.0057 (6)
C16	0.0433 (10)	0.0562 (11)	0.0336 (7)	−0.0003 (8)	0.0043 (7)	0.0085 (7)
C17	0.0450 (10)	0.0574 (11)	0.0337 (7)	−0.0060 (9)	0.0079 (7)	−0.0058 (7)
C18	0.0543 (11)	0.0395 (9)	0.0419 (8)	−0.0010 (8)	0.0101 (8)	−0.0079 (7)
C19	0.0490 (10)	0.0371 (9)	0.0331 (7)	0.0037 (7)	0.0064 (7)	0.0001 (6)
C20	0.0766 (16)	0.0857 (17)	0.0393 (9)	−0.0105 (13)	0.0040 (10)	−0.0183 (10)
C21	0.0660 (13)	0.0320 (8)	0.0312 (7)	−0.0041 (8)	0.0037 (8)	−0.0015 (6)
C22	0.0586 (13)	0.0430 (11)	0.0522 (10)	−0.0016 (9)	−0.0009 (9)	−0.0016 (8)
C23	0.0576 (14)	0.0649 (15)	0.0721 (14)	0.0066 (11)	−0.0098 (11)	−0.0138 (12)
C24	0.0903 (16)	0.0486 (13)	0.0683 (14)	0.0214 (12)	−0.0207 (12)	−0.0075 (11)
C25	0.1190 (19)	0.0403 (12)	0.0612 (13)	0.0079 (13)	0.0006 (13)	0.0060 (10)
C26	0.0971 (18)	0.0356 (10)	0.0508 (10)	−0.0017 (11)	0.0168 (11)	0.0063 (8)

### Geometric parameters (Å, °)

**Table:**

S1—C4	1.7401 (16)	C14—C15	1.386 (2)
S1—C21	1.765 (2)	C14—C19	1.388 (2)
O1—N12	1.217 (2)	C15—C16	1.383 (2)
O2—N12	1.214 (2)	C15—H15	0.9300
O3—N13	1.214 (3)	C16—C17	1.377 (3)
O4—N13	1.205 (3)	C16—H16	0.9300
N1—C5	1.3519 (19)	C17—C18	1.382 (3)
N1—N2	1.3741 (16)	C17—C20	1.505 (2)
N1—C6	1.399 (2)	C18—C19	1.381 (2)
N2—C3	1.319 (2)	C18—H18	0.9300
C3—C4	1.4223 (19)	C19—H19	0.9300
C3—C14	1.470 (2)	C20—H20A	0.9600
C4—C5	1.354 (2)	C20—H20B	0.9600
C5—H5	0.9300	C20—H20C	0.9600
C6—C11	1.389 (2)	C21—C22	1.368 (3)
C6—C7	1.399 (2)	C21—C26	1.391 (3)
C7—C8	1.374 (3)	C22—C23	1.387 (3)
C7—N12	1.470 (2)	C22—H22	0.9300
C8—C9	1.366 (3)	C23—C24	1.376 (4)
C8—H8	0.9300	C23—H23	0.9300
C9—C10	1.368 (3)	C24—C25	1.356 (4)
C9—N13	1.467 (3)	C24—H24	0.9300
C10—C11	1.373 (3)	C25—C26	1.365 (4)
C10—H10	0.9300	C25—H25	0.9300
C11—H11	0.9300	C26—H26	0.9300
			
C4—S1—C21	102.82 (9)	C19—C14—C3	121.15 (14)
C5—N1—N2	110.95 (12)	C16—C15—C14	120.23 (16)
C5—N1—C6	130.11 (12)	C16—C15—H15	119.9
N2—N1—C6	118.49 (12)	C14—C15—H15	119.9
C3—N2—N1	105.38 (11)	C17—C16—C15	121.31 (16)
N2—C3—C4	110.62 (13)	C17—C16—H16	119.3
N2—C3—C14	120.03 (12)	C15—C16—H16	119.3
C4—C3—C14	129.34 (14)	C16—C17—C18	118.25 (15)
C5—C4—C3	105.32 (13)	C16—C17—C20	121.27 (18)
C5—C4—S1	125.04 (11)	C18—C17—C20	120.48 (19)
C3—C4—S1	129.45 (12)	C19—C18—C17	121.21 (17)
N1—C5—C4	107.69 (13)	C19—C18—H18	119.4
N1—C5—H5	126.2	C17—C18—H18	119.4
C4—C5—H5	126.2	C18—C19—C14	120.21 (15)
C11—C6—C7	117.49 (15)	C18—C19—H19	119.9
C11—C6—N1	117.95 (14)	C14—C19—H19	119.9
C7—C6—N1	124.52 (14)	C17—C20—H20A	109.5
C8—C7—C6	121.46 (16)	C17—C20—H20B	109.5
C8—C7—N12	114.90 (16)	H20A—C20—H20B	109.5
C6—C7—N12	123.46 (15)	C17—C20—H20C	109.5
C9—C8—C7	118.47 (17)	H20A—C20—H20C	109.5
C9—C8—H8	120.8	H20B—C20—H20C	109.5
C7—C8—H8	120.8	C22—C21—C26	119.7 (2)
C8—C9—C10	122.32 (17)	C22—C21—S1	124.62 (14)
C8—C9—N13	118.6 (2)	C26—C21—S1	115.67 (17)
C10—C9—N13	119.12 (19)	C21—C22—C23	119.4 (2)
C9—C10—C11	118.73 (18)	C21—C22—H22	120.3
C9—C10—H10	120.6	C23—C22—H22	120.3
C11—C10—H10	120.6	C24—C23—C22	120.2 (3)
C10—C11—C6	121.46 (17)	C24—C23—H23	119.9
C10—C11—H11	119.3	C22—C23—H23	119.9
C6—C11—H11	119.3	C25—C24—C23	120.0 (2)
O2—N12—O1	124.99 (19)	C25—C24—H24	120.0
O2—N12—C7	117.12 (19)	C23—C24—H24	120.0
O1—N12—C7	117.82 (15)	C24—C25—C26	120.7 (2)
O4—N13—O3	124.1 (2)	C24—C25—H25	119.6
O4—N13—C9	118.2 (2)	C26—C25—H25	119.6
O3—N13—C9	117.8 (2)	C25—C26—C21	120.0 (3)
C15—C14—C19	118.75 (14)	C25—C26—H26	120.0
C15—C14—C3	120.07 (14)	C21—C26—H26	120.0
			
C5—N1—N2—C3	−1.74 (18)	C8—C7—N12—O2	34.3 (2)
N2—N1—N2—C3	0(64)	C6—C7—N12—O2	−150.55 (18)
C6—N1—N2—C3	−174.80 (14)	C8—C7—N12—O1	−142.78 (18)
N1—N2—C3—C4	0.97 (18)	C6—C7—N12—O1	32.4 (3)
N1—N2—C3—C14	−178.58 (14)	C8—C9—N13—O4	172.7 (2)
N2—C3—C4—C5	0.11 (19)	C10—C9—N13—O4	−6.6 (3)
C14—C3—C4—C5	179.60 (16)	C8—C9—N13—O3	−7.0 (3)
N2—C3—C4—S1	175.28 (13)	C10—C9—N13—O3	173.7 (2)
C14—C3—C4—S1	−5.2 (3)	N2—C3—C14—C15	−39.9 (2)
C21—S1—C4—C5	−89.59 (16)	C4—C3—C14—C15	140.64 (18)
C21—S1—C4—C3	96.11 (16)	N2—C3—C14—C19	138.13 (17)
N2—N1—C5—C4	1.85 (19)	C4—C3—C14—C19	−41.3 (3)
C6—N1—C5—C4	173.86 (16)	C19—C14—C15—C16	−1.8 (2)
C3—C4—C5—N1	−1.17 (18)	C3—C14—C15—C16	176.31 (15)
S1—C4—C5—N1	−176.61 (12)	C14—C15—C16—C17	0.3 (3)
C5—N1—C6—C11	−148.40 (17)	C15—C16—C17—C18	1.1 (3)
N2—N1—C6—C11	23.1 (2)	C15—C16—C17—C20	−178.87 (18)
C5—N1—C6—C7	29.1 (3)	C16—C17—C18—C19	−1.0 (3)
N2—N1—C6—C7	−159.39 (15)	C20—C17—C18—C19	178.95 (19)
C11—C6—C7—C8	2.8 (2)	C17—C18—C19—C14	−0.5 (3)
N1—C6—C7—C8	−174.74 (16)	C15—C14—C19—C18	1.9 (3)
C11—C6—C7—N12	−172.09 (16)	C3—C14—C19—C18	−176.21 (16)
N1—C6—C7—N12	10.4 (3)	C4—S1—C21—C22	−7.65 (17)
C6—C7—C8—C9	−3.0 (3)	C4—S1—C21—C26	173.29 (14)
N12—C7—C8—C9	172.26 (17)	C26—C21—C22—C23	0.5 (3)
C7—C8—C9—C10	0.9 (3)	S1—C21—C22—C23	−178.52 (15)
C7—C8—C9—N13	−178.39 (18)	C21—C22—C23—C24	−0.9 (3)
C8—C9—C10—C11	1.3 (3)	C22—C23—C24—C25	0.5 (3)
N13—C9—C10—C11	−179.38 (18)	C23—C24—C25—C26	0.4 (4)
C9—C10—C11—C6	−1.5 (3)	C24—C25—C26—C21	−0.8 (3)
C7—C6—C11—C10	−0.5 (3)	C22—C21—C26—C25	0.4 (3)
N1—C6—C11—C10	177.22 (17)	S1—C21—C26—C25	179.46 (17)

### Hydrogen-bond geometry (Å, °)

**Table:**

*D*—H···*A*	*D*—H	H···*A*	*D*···*A*	*D*—H···*A*
C5—H5···N2^i^	0.93	2.44	3.2847 (18)	152

Symmetry codes: (i) *x*, −*y*+1/2, *z*+1/2.
